# Thermal Change Index-Based Diabetic Foot Thermogram Image Classification Using Machine Learning Techniques

**DOI:** 10.3390/s22051793

**Published:** 2022-02-24

**Authors:** Amith Khandakar, Muhammad E. H. Chowdhury, Mamun Bin Ibne Reaz, Sawal Hamid Md Ali, Tariq O. Abbas, Tanvir Alam, Mohamed Arselene Ayari, Zaid B. Mahbub, Rumana Habib, Tawsifur Rahman, Anas M. Tahir, Ahmad Ashrif A. Bakar, Rayaz A. Malik

**Affiliations:** 1 Department of Electrical Engineering, Qatar University, Doha 2713, Qatar; amitk@qu.edu.qa (A.K.); tawsifur.rahman@qu.edu.qa (T.R.); a.tahir@qu.edu.qa (A.M.T.); 2 Department of Electrical, Electronic and Systems Engineering, Faculty of Engineering and Built Environment, Universiti Kebangsaan Malaysia, Bangi 43600 UKM, Selangor, Malaysia; sawal@ukm.edu.my (S.H.M.A.); ashrif@ukm.edu.my (A.A.A.B.); 3 Urology Division, Surgery Department, Sidra Medicine, Doha 26999, Qatar; tabbas-c@sidra.org; 4 College of Science and Engineering, Hamad Bin Khalifa University, Doha 34110, Qatar; talam@hbku.edu.qa; 5 College of Engineering, Qatar University, Doha 2713, Qatar; arslana@qu.edu.qa; 6 Department of Mathematics and Physics, North South University, Dhaka 1000, Bangladesh; zaid.mahbub@northsouth.edu; 7 Neurology Department, BIRDEM General Hospital, Dhaka 1000, Bangladesh; drhrumana4@gmail.com; 8 Weill Cornell Medicine-Qatar, Ar-Rayyan 24144, Qatar; ram2045@qatar-med.cornell.edu

**Keywords:** diabetic foot, thermogram, thermal change index, machine learning, deep learning

## Abstract

Diabetes mellitus (DM) can lead to plantar ulcers, amputation and death. Plantar foot thermogram images acquired using an infrared camera have been shown to detect changes in temperature distribution associated with a higher risk of foot ulceration. Machine learning approaches applied to such infrared images may have utility in the early diagnosis of diabetic foot complications. In this work, a publicly available dataset was categorized into different classes, which were corroborated by domain experts, based on a temperature distribution parameter—the thermal change index (TCI). We then explored different machine-learning approaches for classifying thermograms of the TCI-labeled dataset. Classical machine learning algorithms with feature engineering and the convolutional neural network (CNN) with image enhancement techniques were extensively investigated to identify the best performing network for classifying thermograms. The multilayer perceptron (MLP) classifier along with the features extracted from thermogram images showed an accuracy of 90.1% in multi-class classification, which outperformed the literature-reported performance metrics on this dataset.

## 1. Introduction

Diabetes mellitus (DM) is characterized by hyperglycemia which can lead to pathology in the brain, heart, eyes, kidney’s and lower limbs [1]. DM leads to diabetic foot ulceration (DFU), which may not heal adequately due to poor microvascular and macrovascular tissue perfusion and infection and may eventually lead to lower limb amputation [2,3]. Early detection and better classification of foot complications may enable timely intervention and effective treatment to either heal foot ulcers or prevent progression to amputation. Early monitoring by self-diagnosis at home could be useful in preventing the development and progression of DFU. However, the easiest monitoring technique, visual inspection, has its limitations, for example, people with obesity or visual impairment cannot adequately detect early changes. According to recent studies, a home temperature monitoring system could detect 97% of diabetic foot ulcers (DFUs) well in advance [4,5,6,7]. Patients undergoing continuous temperature monitoring of their feet have a lower risk of foot complications [8]. 

Non-invasive thermographic imaging using an infrared (IR) camera are popular techniques to examine for thermal changes in images [9] and have been used to detect thermal changes in the diabetic foot [10]. The analysis is based on the intensity of the infrared light emitted or reflected by the tissue based on the controllable excitation used during the imaging of infrared rays. Thermal infrared imaging-based machine vision (IRMV) can be categorized into passive thermography and active thermography [9]. Passive thermography is used for the human body, which is self-heating unlike active thermography, which is used in non-self-heating objects. Several studies have proposed thermogram-based techniques for the study of the diabetic foot, where they reported that the control group showed a specific butterfly pattern, while the DM group showed a large variety of spatial patterns [11]. One can calculate and estimate thermal changes comparing the contralateral foot as a reference comparison for temperature [12,13,14,15]. However, if both feet have temperature changes, but none have the butterfly pattern, one foot cannot act as a reference [16,17,18]. Thus, self-diagnosis at home will require a medical experts’ opinion.

Machine learning (ML) techniques are gaining popularity in biomedical applications to assist the medical expert in early diagnosis [19,20,21]. Several studies [17,22,23,24] have attempted to extract features that can be used to identify the hot region in the plantar thermogram, which could be a sign of tissue damage or inflammation (details are provided later). We have previously trained an AdaBoost classifier, which achieved an F1-score of 97% in classifying diabetic and healthy patients using thermogram images [23]. The features used in our previous study [23] are provided in detail in a later section of this paper. Hernandez et al. [17] proposed a quantitative index called the thermal change index (TCI) for measuring the thermal change in the plantar region of diabetic patients in comparison to the reference control group and then use the TCI to automatically classify the patients. Hernandez et al. [17,22] shared a public database called the “Plantar Thermogram Database” of foot thermogram images from control and diabetic patients and used the TCI to classify the subjects into five classes (Class 1 to Class 5) depending on the spatial temperature distribution and the range of temperatures. Cruz-Vega et al. [24] proposed a deep learning technique to classify the images of that database in a non-conventional classification scheme, where the results were shown by taking two classes at a time and then averaging the results after ten-fold cross-validation of a different combination of 2 set classes. A new diabetic foot thermogram network (DFTNet) was proposed for the classification of Class 3 and Class 4 with a sensitivity and accuracy of 0.9167 and 0.853, respectively [24]. 

Despite this earlier work, there is still a need to improve the level of machine learning performance for the early detection of diabetic foot ulceration. This has motivated this detailed investigation. The manuscript is organized into five sections: Section 1 is the introduction and related works; Section 2 discusses the research significance and highlights the key contributions; Section 3 discusses the detailed methodology, and Section 4 and Section 5 present the results and discussion. Finally, Section 6 presents the conclusions.

## 2. Research Significance

The importance of the early detection of diabetic foot problems and the gaps in terms of machine learning performance accuracy were the major research questions of this paper. The non-conventional classification scheme used in recent work [23] and the moderate performance of existing machine-learning models [24] motivated us to investigate and propose a generic framework for the multi-class (*n* = 5) classification of thermogram images while enhancing the classification performance further. We investigated classical ML techniques with feature engineering and convolutional neural network (CNN)-based models with image enhancement techniques to identify the best performing classification model. To the best of our knowledge, this is the best reported performance for the classification of foot thermograms into different classes (based on TCI). The major contributions of this paper are highlighted below:

- Extraction and ranking of the relevant features from the temperature pixels for classifying the thermogram images into TCI-based classes.
- Explores the effect of various image enhancement techniques on thermogram images in improving the performance of 2D CNN models in TCI-based classes.
- Investigation of different ML classifiers with feature engineering for enhanced classification performance.
- Proposes a machine-learning framework that outperforms the DFTNet by a significant margin in classifying thermograms into TCI-based classes.

## 3. Methodology

**Figure 1:**
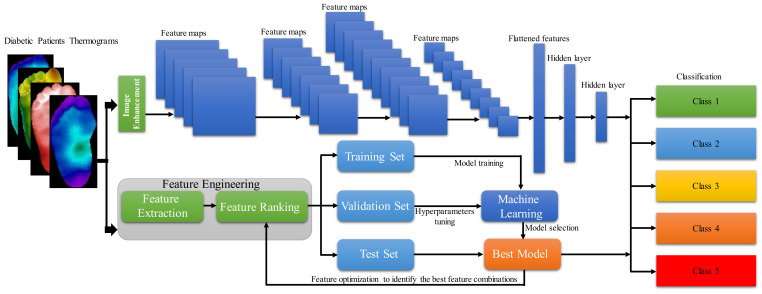
Illustration of the computational workflow for this study.

Figure 1 summarizes the overall methodology adopted for this study, where the thermogram is applied as input to the different 2D CNN models using different image enhancement techniques [25] and classical ML algorithms on the extracted features from the thermograms [23]. The section below discusses in detail the dataset used for the study and the details of the investigation done using (i) thermogram images by the 2D CNN models along with different image enhancement techniques and (ii) classical ML algorithms with feature engineering (feature extraction, and feature reduction). This section also provides details of the performance metrics used for identifying the best-performing machine-learning algorithm.

### 3.1. Dataset Description

**Figure 2:**
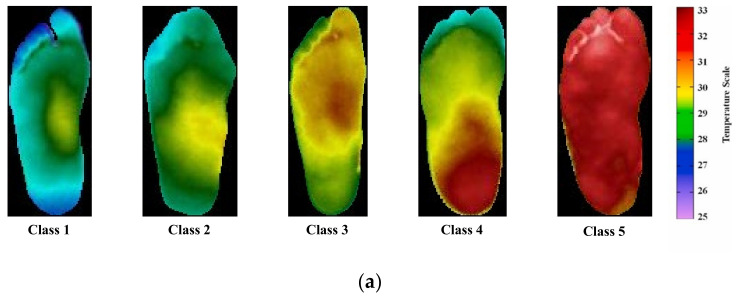

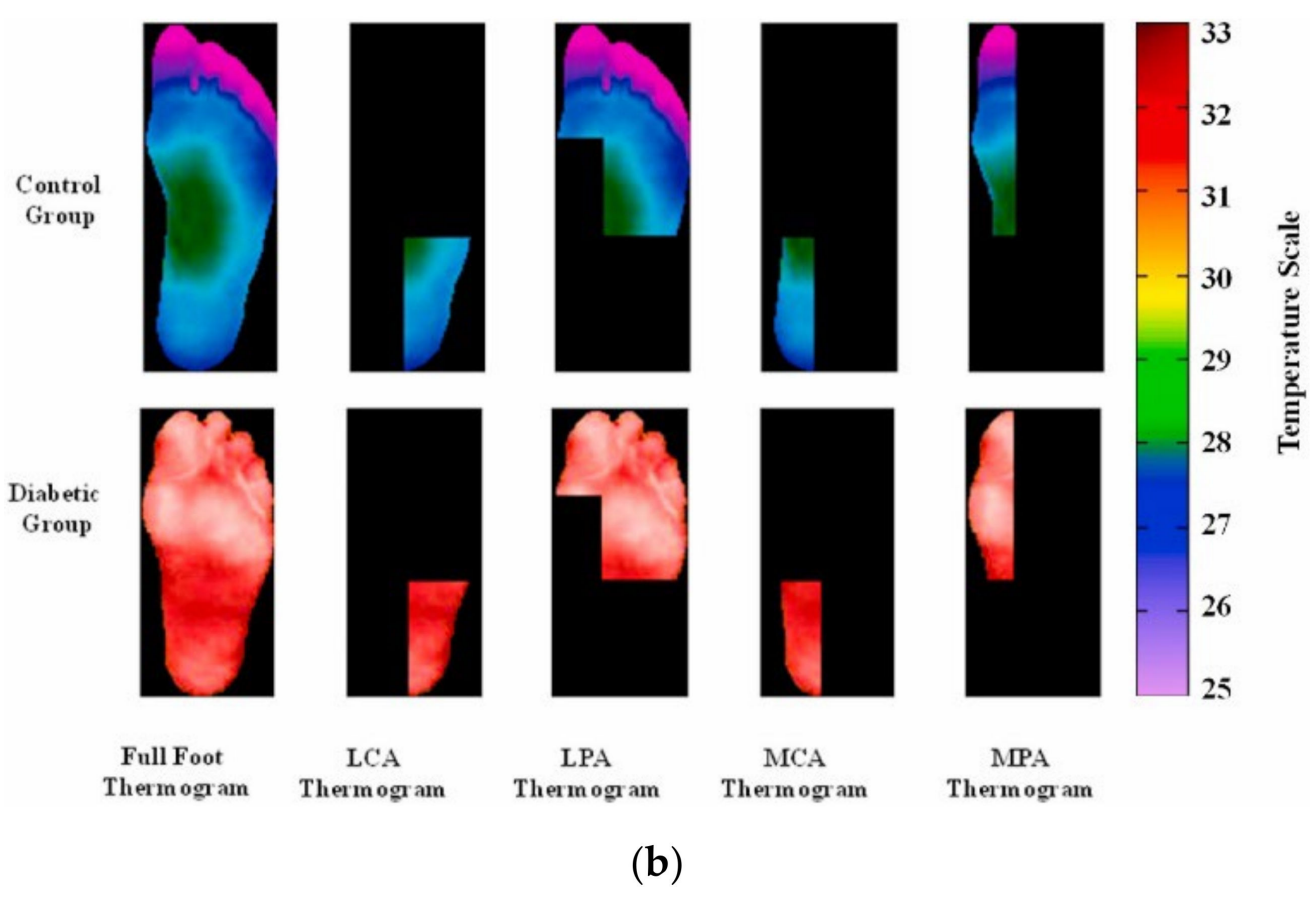
(**a**) Sample of thermograms from different classes [22] and (**b**) sample of MPA, LPA, MCA, and LCA angiosomes of the foot for the control and diabetic Group [23].

In this study, 122 foot-pair thermograms of DM patients obtained from a public thermogram database [22] were used. The dataset contained demographic information such as age, gender, height, and weight of the patients/participants. The dataset was grouped into five different classes (Class 1, Class 2, Class 3, Class 4, and Class 5) based on the thermal change index (TCI) which is defined as

(1) $$Thermal\;Change\;Index\;\left(TCI\right)=\frac{CG_{ang}-DM_{ang}\;}{4}\;\;\;\;\;\;\;\;\;\;\;\;\;\;\;\;\;\;\;$$

where *CG_ang_* and *DM_ang_* are the temperature values of the angiosome for the control and subjects with diabetes, respectively. The TCI values for different classes can be seen in Table 1. Examples of thermogram images classified into five classes are shown in Figure 2a. 

The dataset also provided the segmented thermograms of four angiosomes: the medial plantar artery (MPA), lateral plantar artery (LPA), medial calcaneal artery (MCA), and lateral calcaneal artery (LCA) (Figure 2b). The concept of four angiosomes was proposed by Taylor and Palmer [26] and they provide valuable information related to the damage generated by DM in arteries as well as the associated ulceration risk since it is used to compute the local temperature distribution. The dataset provided the pixelated temperature readings for the full foot and the four angiosomes for both feet. The emissivity settings in the acquiring camera were set to 0.98, which is the emissivity of human skin [27]; objects with emissivities higher than 0.5 do not usually suffer from inaccurate temperature measurements when using an IR camera [28].

### 3.2. Image Pre-Processing

**Figure 3:**
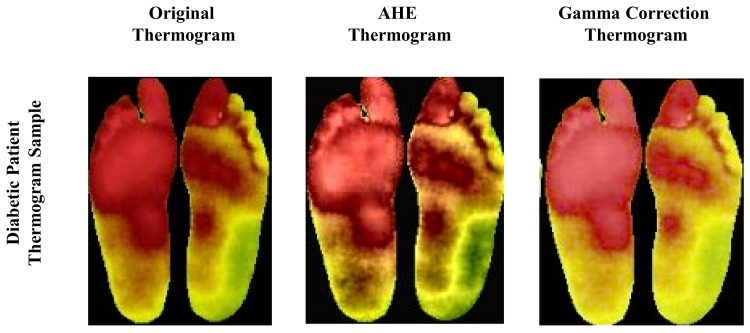
Original thermogram versus enhanced thermogram using AHE and Gamma correction for diabetic patient’s thermogram [23].

Image enhancement techniques such as adaptive histogram equalization (AHE) [29] and Gamma correction [25] may help improve the classification of thermograms [23]. Thus, for the original images, we also generated AHE and Gamma-enhanced thermogram images. Some examples of the image enhancement on the thermograms can be seen in Figure 3. 

#### D CNN-Based Classification

The application of 2D CNN in biomedical image-domain applications is very popular for automatic and early detection of abnormalities such as COVID-19 pneumonia [25,30,31], tuberculosis [32], community-acquired pneumonia [33], and many others [34]. A labeled dataset can be divided into training and testing datasets, where the training dataset is used to train the network and its performance is verified by the unseen test set. A part of the training dataset is used for validation during the training process, which is used to avoid overfitting [25,30,31,32,33]. In this study, five-fold cross-validation was used, i.e., the dataset was divided into five-folds, and the confusion matrices for the test set of each fold were combined to calculate the performance metrics of the entire dataset. The overall accuracy and weighted metrics such as the precision, recall, specificity, and F1-score were calculated. As a large dataset is required to train a deep learning model to avoid a model over-fitting problem, popular augmentation techniques (i.e., rotation and translation) were used to increase the training data size [25,30,31,32,33]. The details of the training, validation, and testing sets for the 5-class image dataset labeled using TCI [22], are shown in Table 1.

As we had a limited number of images in the dataset (Table 1), we used pre-trained models that were already pre-trained on a large ImageNet database [35]. These pre-trained networks have a good performance on the ImageNet database and can be further trained for our problem by fine-tuning the deep learning models using our dataset. Based on an extensive literature review and previous performances [25,30,31,32,33], in this study, six well-known pre-trained deep CNN models were used for the thermograms’ classification: ResNet18, ResNet50 [36], DenseNet201 [36], InceptionV3 [37], VGG19 [38] and MobileNetV2 [39]. All the above-mentioned six CNN-based models were trained, validated, and tested on the original, AHE, and Gamma-enhanced thermogram images and performance metrics were calculated after five-fold cross-validation to identify the best network and image enhancement technique combination. 

### 3.3. Classical Machine Learning Approach 

This section discusses the features extracted from the thermograms, feature reduction techniques, feature ranking techniques, classical ML models, and details of our extensive investigations.

#### 3.3.1. Feature Extraction and Reduction:

We carefully reviewed the literature to summarize the features that have been used in clinical practice and ML approaches to analyze foot thermograms for the diagnosis of the diabetic foot. The details of the final list of features identified can be found in our previous work [23] and are discussed briefly below:

(2) $$Estimated\;Temperature\;\left(ET\right)=\;\frac{a_{j-1}C_{j-1\;}+a_{j}C_{j}+a_{j+1}C_{j+1}}{a_{j-1\;}+a_{j}+a_{j+1\;}}$$

(3) $$Estimated\;Temperature\;Difference\;\left(ETD\right)=\;|ET_{left\;Angiosome}-ET_{right\;angiosome}|\;\;\;$$

(4) $$Hot\;Spot\;Estimator\;\left(HSE\right)=|c_{l}-ET|$$

The term *C_j_* and *a_j_* denote the classmark temperature and the corresponding percentage of pixels in that region, respectively. The values *a_j−1_* and *a_j+1_* are the percentage of pixels in the neighboring classmark temperatures, *C_j−1_* and *C_j+1_*, respectively. To equate the parameters in Equations (2)–(4), a histogram was generated for the percentage of pixels in the thermogram (either full foot or angiosomes) for the different classmark temperatures (C_0_ = 26.5 °C, C_1_ = 28.5 °C, C_2_ = 29.5 °C, C_3_ = 30.5 °C, C_4_ = 31 °C, C_5_ = 32.5 °C, C_6_ = 33.5 °C, C_7_ = 34.5 °C). 

Statistical parameters such as the mean, standard deviation, and median are very important features in various ML approaches for biomedical applications [40,41,42,43]; these were calculated as well. In addition to these parameters, we formulated several parameters that are visually very important to distinguish the variation in the plantar temperature distribution, such as the normalized range temperature for class *j (NRTclass j),* which were also reported in our previous work [23]. The variable *NRTclass j* is the number of pixels in *class j* temperature range over the total number of non-zero pixels, where *class j* can be class 1 to 5. For the temperature ranges in the class, we have used the same temperature range as reported in [22]. 

Finally, we summarized a total of 39 features that can be used for the early detection of the diabetic foot, which are Age, Gender, TCI, Highest Temperature value, NRT (Class 1–5), HSE, ET, ETD, Mean, Median, SD of temperature for the different angiosomes LPA, LCA, MPA, MCA and for Full Foot. We have previously reported the statistics for the data along with the source code [22] in our previous work [23].

The final list of features was optimized by removing redundant features based on the correlation between different features. Features with more than a 95% correlation were removed, which improves the overall performance by reducing the number of redundant features by avoiding overfitting [41,42,43,44].

#### 3.3.2. Feature Ranking

Providing the ML classifier with a large number of features could lead to overfitting and lower performance as the excess information might provide contradictory details and confuse the classifier [23,45,46,47,48]. With the help of feature ranking techniques, the classifiers can be provided with the important features and their performance can be checked accordingly. This process can help to finalize the features to be used as input to the ML classifiers. In this paper, we used state-of-the-art and popular feature ranking techniques based on ML algorithms—XGBoost [49], Random Forest [50], and Extra Tree [51]. These feature ranking techniques have proven to be very useful for different biomedical applications [47,48,52,53].

#### 3.3.3. Classical Machine Learning Models

After the feature extraction, feature reduction, and feature ranking, different classical ML models were investigated to compare the performances. Data in different classes were imbalanced, and therefore, to avoid imbalanced training datasets and biased results, the popular synthetic minority oversampling technique (SMOTE) [54] was used to make them balance. The ten popular ML classifiers used in the study were multilayer perceptron (MLP) [55], Support Vector Machine(SVM) [56], Random Forest [50], Extra Tree [51], GradientBoost [57], Logistic regression [58], K Nearest Neighbor (KNN) [59], XGBoost [49], AdaBoost [60], and Linear Discriminant Analysis (LDA) [61]. Amongst the state of the art and popular machine learning models, the XGBoost, Random Forest, and Extra Tree machine learning networks have been popular in recent clinical applications. 

Multi-Tree Extreme Gradient Boosting (XGBoost) has been frequently applied for feature selection because of its speed, efficiency, and scalability [62]. The importance of each feature in XGBoost is determined by its accumulated use in each decision step in trees. This computes a metric that characterizes the relative importance of each feature, which is particularly valuable to estimate features that are the most discriminative of model outcomes, especially when they are related to meaningful clinical parameters.

Random Forests are often used for feature selection in machine learning because the tree-based strategies used by random forests naturally rank by how well they improve the purity of the node. Nodes with the greatest decrease in impurity happen at the start of the trees, while nodes with the least decrease in impurity occur at the end of trees. Thus, by pruning trees below a particular node, we can create a subset of the most important features [50].

Moreover, Extra Tree is a model-based approach for selecting the features using the tree-based supervised models to make decisions on the importance of the features. The Extra Tree classifier or the Extremely Random Tree Classifier is an ensemble algorithm that seeds multiple tree models constructed randomly from the training dataset and sorts out the features that have been most voted for. It fits each decision tree on the whole dataset rather than a bootstrap replica and picks out a split point at random to split the nodes. The splitting of nodes that occurs at every level of the constituent decision trees is based on the measure of randomness or entropy in the sub-nodes. The nodes are split on all variables available in the dataset, and the split that results in the most homogenous sub-child is selected in the constituent tree models. This lowers the variance and makes the model less prone to overfitting [51].

### 3.4. Performance Evaluation

In all of our experiments, we reported the sensitivity, specificity, precision, accuracy, F1-score, and area under the curve (AUC) for five-folds as our evaluation metrics. It is well known that in multi-class classification applications, sensitivity and specificity are relevant metrics to evaluate a classifier’s performance [63]:

(5) $$Accuracy_{class\_i}=\frac{TP_{class\_i}+TN_{class\_i}}{TP_{class\_i}+TN_{class\_i}+FP_{class\_i}+FN_{class\_i}}\;$$

(6) $$Precision_{class\_i}=\frac{TP_{class\_i}}{TP_{class\_i}+FP_{class\_i}}\;$$

(7) $$Sensitivity_{class_{i}}=\frac{TP_{class_{i}}}{TP_{class_{i}}+FN_{class_{i}}}\;$$

(8) $$F1\_score_{class_{i}}=2\frac{Precision_{class_{i}}\times Sensitivity_{class_{i}}}{Precision_{class_{i}}+Sensitivity_{class_{i}}}\;$$

(9) $$Specificity_{class\_i}=\frac{TN_{class\_i}}{TN_{class\_i}+FP_{class\_i}}\;$$

where $\;class_{i}=Class\;1,\;Class\;2,\;Class\;3,\;Class\;4\;and\;Class\;5$.

Here, *TP*, *FP, TN*, and *FN* are true positive, false positive, true negative, and false negative, respectively. Here, *TP* is the number of correctly identified thermograms in a particular class_i_, *TN* is the number of correctly identified thermograms of the other classes, FP is the number of thermograms misclassified to class_i_ and *FN* is the number of thermograms of class_i_ incorrectly classified to other classes. The weighted performance metrics, with a 95% confidence interval, for sensitivity, specificity, precision, and F1-score, were reported and for the accuracy, the overall accuracy, with a 95% confidence interval, was reported. 

All the experiments were done with a computer with Intel i7–10750H @2.6 GHz CPU, NVIDIA GeForce RTX 2070 Super GPU, 32 GB RAM. Python, Matlab, and Stata/MP 13.0 software were used for the study. 

## 4. Experimental Results

This section provides the results of the various experiments in this study. 

### 4.1. D CNN-Based Classification

**Figure 4:**
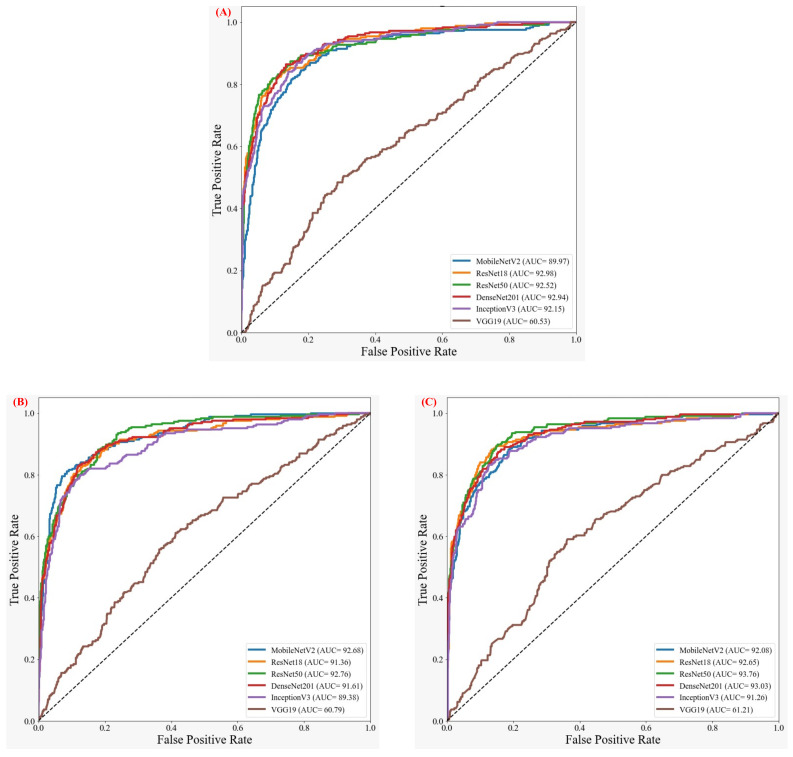
AUC for the (**A**) original, (**B**) AHE-enhanced, and (**C**) Gamma-enhanced thermograms in TCI-based classification.

As discussed earlier, the authors investigated six pre-trained networks (ResNet18, ResNet50, VGG19, DenseNet201, InceptionV3, and MobileNetV2), along with popular image enhancement techniques. AHE did not improve the performance for different networks compared to the original whereas GAMMA correction helped in sharpening the distinguishing features. Independent foot images were used to check if the different pre-trained networks could classify them into different classes or not. Table 2 reports the five best performing combinations of network and enhancement type and it shows that the performance was not that promising after an extensive investigation using popular 2D CNN networks and popular image enhancement techniques. The results can be further analyzed using the AUCs for the original, AHE, and GAMMA correction thermogram as shown in Figure 4. 

### 4.2. Classical Machine Learning-Based Classification

Since the above investigation showed that the different 2D CNN models and image enhancement techniques did not adequately distinguish different image classes, particularly Classes 2–4, the authors investigated the classical ML models using feature engineering to assess their performance. Thirty-eight features were extracted as discussed earlier from the thermograms images from different classes. These 38 features were optimized to remove redundant features by finding the correlation between the different features. Features with more than 95% correlation were removed, resulting in 28 features. The heat maps of the correlation matrix with all features and after removing the highly correlated features are shown in Supplementary Materials, Figure S1. The resultant 28 features were Gender; Age; NRT (Class 1); NRT (Class 2); NRT (Class 3); NRT (Class 4); NRT (Class 5); Highest Temperature; HSE, ETD, and STD of MPA; HSE, ET, ETD, Mean, STD of LPA; HSE, ET, ETD, Mean, STD of LCA; HSE, ET, ETD, STD of MCA; HSE, ETD, STD of Full foot. 

**Figure 5:**
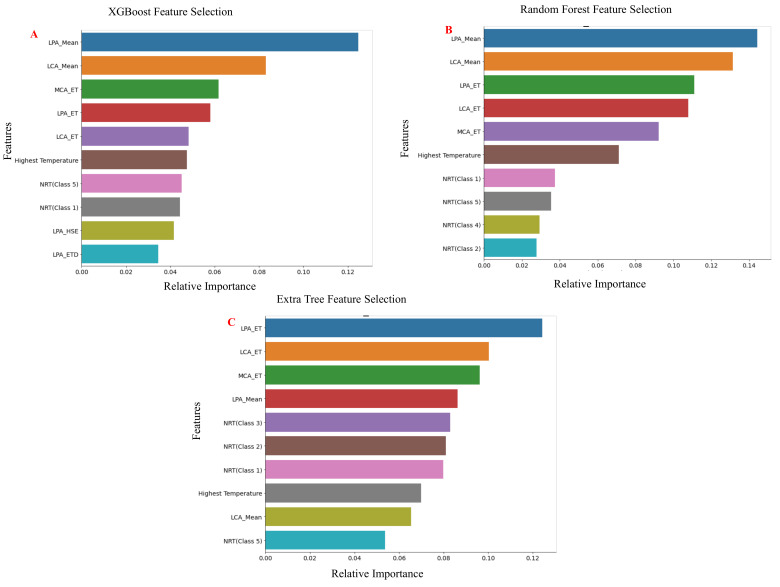
Top ranked 10 features from the reduced 28 features using feature selection techniques (**A**) XGBoost, (**B**) Random Forest, and (**C**) Extra Tree.

In this experiment, three feature selection techniques (Extra Tree, XGBoost, and Random Forest) with 10 machine-learning models were investigated with 28 optimized features to identify the best combination using 810 different investigations. The top-ranked 10 features using the three different feature-ranking techniques can be seen in Figure 5. 

The overall performance for the top-performing combination (feature ranking and features) using ten classical ML classifiers and the detailed class-wise summary of the top-performing combination amongst them is presented in Table 3 and Table 4, respectively. It can be seen that the MLP classifier with the XGboost feature selection technique and the top two features (mean of LPA and LCA) shows the best performance of 91.18% (weighted F1-score) in the stratification of the thermogram into different classes (1 to 5) using the TCI.

## 5. Discussion

To the best of our knowledge, no previous study has investigated TCI-based diabetic foot classification for five-class stratification using 2D CNNs and using the original and enhanced thermogram images. Different pre-trained networks were investigated and we found that the image enhancement techniques did not help much in the classification performance. The ROC curves in Figure 5 confirm that the vanilla CNN architecture (such as VGG-19) performed worse in the classification, even with the image enhancement techniques. Other complex networks such as networks with residual connections (ResNet18, ResNet50), networks with concatenations (DenseNet201), networks with inception blocks (InceptionV3), and networks with inverted residual and linear bottleneck layers (MobileNetV2) performed relatively better than VGG 19, but the individual class-wise performance was not acceptable. Image enhancement techniques such as AHE degraded the performance but Gamma correction provided a performance similar to the original thermograms. Gamma correction helped in making the distinguishable features more evident, which was also evident in similar other studies [23]. A better understanding of the machine-learning performance can be analyzed using the F1-score, which is calculated using precision and sensitivity, especially for multiclass problems [64]. The best performing combination was the ResNet50 network using the original thermogram images with a weighted F1-score of 76.66%, followed by the MobileNetv2 network (75.74%) using the AHE-enhanced thermogram, ResNet18 using the original thermogram (75.61%), and ResNet18 and ResNet50 using the Gamma-enhanced thermogram provided scores of 74.41%, and 74.17%, respectively. It can be further seen in Table 2 that the weighted F1-score for the top-performing combination, ResNet50 using original thermogram was reasonable for extreme classes (84.48% and 91.02% for the extreme Class 1 and Class 5 categories, respectively, but it was poor for the remaining classes (64.40%, 52.18%, and 60.98% for Class 2, Class 3, and Class 4, respectively). It can be assumed that the middle categories are very similar and thus cannot be easily distinguished by 2D CNN models from the thermogram images. 

This prompted further investigation using the classical ML approach. Interestingly, the novel features extracted by the authors helped to produce better image class stratification compared to the 2D CNN-based deep learning models. The top ten features were identified from the reduced features (28 features after reduction) using different feature selection techniques (XGBoost, Random Forest, and Extra Tree) and are shown in Figure 5. The best-performing feature selection techniques were XGBoost and Random Forest (Table 3) and they identified almost the same top eight features (Mean, ET of LPA, LCA, and ET of MCA, Highest Temperature, NRT (Class 1), and NRT (Class 5). The demographic features are not included in the top 10 features, which means the user demography is independent and the decisions are based only on the temperature information from the foot thermograms. The top 10 features also confirm the importance of LPA and the statistical information (ET, HSE, and Mean) in the classification of diabetic thermograms. The feature proposed by the authors, i.e., NRT, which was developed to find the normalized number of pixels in the distinguishable temperature range of the different classes, can also be useful for classification by the classical ML models. As can be seen in Table 4, the MLP classifier achieved around 88%, 84%, and 83% of F1-score for Class 2, Class 3, and Class 4, respectively, in addition to providing an F1-score of more than 95% for the extreme classes (Class 1 and Class 5).

**Figure 6:**
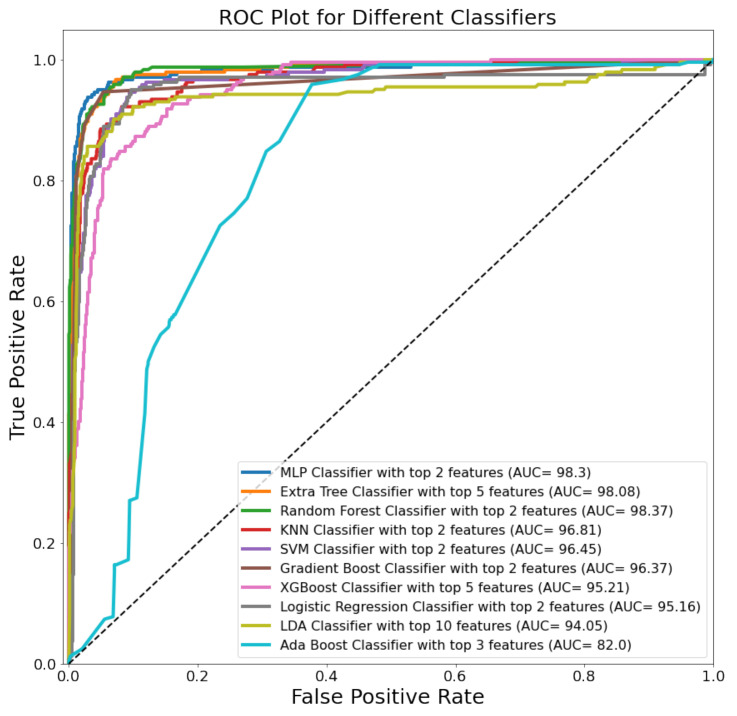
ROC plot for the top-performing feature combination from 10 classical ML classifiers.

**Figure 7:**
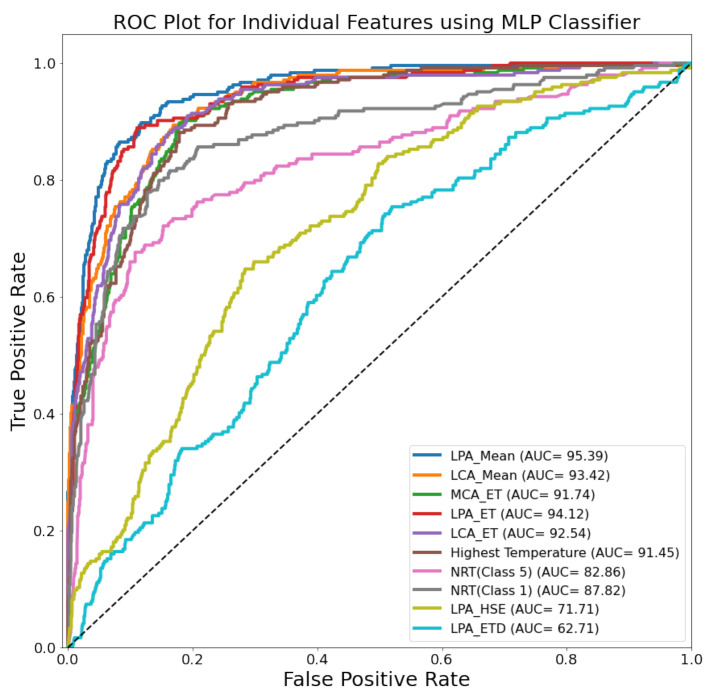
ROC plot for 10 individual features using MLP classifier (best performing model).

Figure 6 shows the ROC curves for the top-performing combination of features and feature-ranking techniques for 10 classifiers, where the MLP outperformed the other ML classifiers. The ROC plot for the individual top 10 features using the MLP classifier can be seen in Figure 7 and the combination of the top two features (mean of LPA and LCA) provided the best results. It seems that they are enough to distinguish the temperature range in the thermogram, which is what the TCI-based classification is based on (Equation (1)). The LPA and LCA are very important angiosomes and have also helped to distinguish control and diabetic patients previously [23].

To the best of the authors’ knowledge, the proposed machine-learning framework is the best performing solution compared to the studies reported in the literature, as summarized in Table 5.

## 6. Conclusions

Diabetic foot is a critical health issue with major ramifications in relation to amputation and mortality. Thus, early detection and severity classification may help to prevent such complications. The deployment of the proposed ML model can help in preparing easy-to-use solutions for early detection; thus, saving the time of medical experts and providing solutions that could be useful for patients in their home settings. Patients can use it at home especially during pandemic situations, when visits to the hospital are limited, avoiding stress on the healthcare system. 

The conclusions drawn from the results in this paper are as follows:

- The relevant features were extracted and ranked from the temperature pixels to classify the thermogram images into TCI-based classes. This is the best reported performance for a machine learning-based foot thermogram classification into different TCI-based classes.
- We explored the effect of various image enhancement techniques on thermogram images to improve the performance of 2D CNN models in TCI-based classes. It was found that the image enhancement techniques did not help to improve the performance, even for the state-of-the-art DFTNet proposed in [24].
- The classical ML classifier’s performance with carefully selected and refined features was exceptionally good compared to the performance of the 2D CNN models with/without image enhancement.
- The proposed machine-learning framework outperforms the DFTNet by a significant margin in classifying thermograms into TCI-based classes. The trained classical ML models can help in the classification using foot thermograms, which can be captured using infrared cameras.

In conclusion, such a system could be easily deployed, and patients could get the benefits of remote healthcare just by using an infrared camera and a mobile application, a future direction of our research. Though the results are promising, it is important to acknowledge some limitations:

- The performance reported uses a publicly available dataset, which has to be further validated for robustness with the help of a new dataset. The authors have already applied to the IRB to collect a new dataset.
- The dataset was collected using two different IR cameras (FLIR E60 and FLIR E6) with different resolutions [22]. However, the trained network is still able to find the distinguishing pattern, which confirms the robustness of different IR cameras, but this needs to be further validated with other IR cameras along with low-resolution IR cameras that are usable with mobile phones.

Nonetheless, the results of this study may facilitate remote health monitoring of diabetic patients from the convenience of their homes. 

## Figures and Tables

**Table 1:** Details of the dataset used for training (with and without augmentation), validation, and testing.

Dataset	Count of Diabetic Thermograms/Cluster Identified in the Paper	Training Dataset Details			
		Training (60% of the Data) Thermogram/Fold	Augmented Train Thermogram/Fold	Validation (20% of the Data) Thermogram/Fold	Test (20% of the Data) Image/Fold
Contreras et al. [22]	Class 1 (TCI < 2)	34	1020	11	11
	Class 2 (2 < TCI < 3)	22	1100	07	07
	Class 3 (3 < TCI < 4)	17	1020	05	06
	Class 4 (4 < TCI < 5)	22	1100	07	08
	Class 5 (5 < TCI)	52	1044	17	18

**Table 2:** Performance metrics and inference time for five-fold cross-validation using 2D CNNs.

Enhancement	Network		95% Confidence Interval Results					
		Class	Accuracy	Precision	Sensitivity	F1-Score	Specificity	Inference Time
Original	ResNet50	Class 1	92.62 ± 06.85	81.67 ± 10.13	87.50 ± 08.66	84.48 ± 09.48	94.15 ± 06.15	6.247
		Class 2	88.93 ± 10.25	60.98 ± 15.93	69.44 ± 15.05	64.40 ± 15.64	92.31 ± 08.70	
		Class 3	90.98 ± 10.61	66.67 ± 17.46	42.86 ± 18.33	52.18 ± 18.50	97.22 ± 06.09	
		Class 4	86.89 ± 10.88	55.56 ± 16.01	67.57 ± 15.08	60.98 ± 15.72	90.34 ± 09.52	
		Class 5	93.85 ± 05.05	95.00 ± 04.58	87.36 ± 06.98	91.02 ± 06.01	97.45 ± 03.31	
		Overall	91.46 ± 03.51	77.69 ± 05.22	76.64 ± 05.31	76.66 ± 05.31	94.83 ± 02.78	
AHE	MobileNetv2	Class 1	94.26 ± 6.09	88.89 ± 08.23	85.71 ± 09.17	87.27 ± 08.73	96.81 ± 04.60	5.412
		Class 2	91.39 ± 09.16	70.27 ± 14.93	72.22 ± 14.63	71.23 ± 14.79	94.71 ± 07.31	
		Class 3	88.11 ± 11.99	47.06 ± 18.49	28.57 ± 16.73	35.55 ± 17.73	95.83 ± 07.40	
		Class 4	84.43 ± 11.68	48.94 ± 16.11	62.16 ± 15.63	54.76 ± 16.04	88.41 ± 10.31	
		Class 5	94.26 ± 04.89	91.01 ± 06.01	93.10 ± 05.33	92.04 ± 05.69	94.90 ± 04.62	
		Overall	91.64 ± 03.47	76.04 ± 05.36	76.23 ± 05.34	75.74 ± 05.38	94.43 ± 02.88	
Original	ResNet18	Class 1	92.21 ± 07.02	83.64 ± 09.69	82.14 ± 10.03	82.88 ± 09.87	95.21 ± 05.59	2.536
		Class 2	88.11 ± 10.57	58.14 ± 16.12	69.44 ± 15.05	63.29 ± 15.75	91.35 ± 09.18	
		Class 3	90.98 ± 10.61	63.64 ± 17.82	50.00 ± 18.52	56.00 ± 18.39	96.30 ± 06.99	
		Class 4	86.89 ± 10.88	56.10 ± 15.99	62.16 ± 15.63	58.97 ± 15.85	91.30 ± 09.08	
		Class 5	92.62 ± 05.49	91.57 ± 05.84	87.36 ± 06.98	89.42 ± 06.46	95.54 ± 04.34	
		Overall	90.80 ± 03.63	76.23 ± 05.34	75.41 ± 05.40	75.61 ± 05.39	94.29 ± 02.91	
Gamma Correction	ResNet18	Class 1	93.03 ± 06.67	88.24 ± 08.44	80.36 ± 10.41	84.12 ± 09.57	96.81 ± 04.60	3.347
		Class 2	89.75 ± 09.91	63.41 ± 15.73	72.22 ± 14.63	67.53 ± 15.30	92.79 ± 08.45	
		Class 3	90.16 ± 11.03	59.09 ± 18.21	46.43 ± 18.47	52.00 ± 18.51	95.83 ± 07.40	
		Class 4	82.79 ± 12.16	44.90 ± 16.03	59.46 ± 15.82	51.16 ± 16.11	86.96 ± 10.85	
		Class 5	91.80 ± 05.77	91.36 ± 05.90	85.06 ± 07.49	88.10 ± 06.80	95.54 ± 04.34	
		Overall	90.23 ± 03.73	75.77 ± 05.38	73.77 ± 05.52	74.41 ± 05.48	94.16 ± 02.94	
Gamma Correction	ResNet50	Class 1	92.21 ± 07.02	80.33 ± 10.41	87.50 ± 08.66	83.76 ± 09.66	93.62 ± 06.40	7.764
		Class 2	88.93 ± 10.25	63.64 ± 15.71	58.33 ± 16.11	60.87 ± 15.94	94.23 ± 07.62	
		Class 3	87.30 ± 12.33	44.00 ± 18.39	39.29 ± 18.09	41.51 ± 18.25	93.52 ± 09.12	
		Class 4	87.70 ± 10.58	59.46 ± 15.82	59.46 ± 15.82	59.46 ± 15.82	92.75 ± 08.36	
		Class 5	93.03 ± 05.35	89.77 ± 06.37	90.80 ± 06.07	90.28 ± 06.22	94.27 ± 04.88	
		Overall	90.77 ± 03.63	73.90 ± 05.51	74.59 ± 05.46	74.17 ± 05.49	93.80 ± 03.03	

**Table 3:** Performance metrics for the best-performing combinations (feature selection technique and number of features) for the 10 ML Classifiers.

Classifier	Feature Selection	# of Feature	95% Confidence Interval Results					Inference Time (ms)
			Accuracy	Precision	Sensitivity	F1-Score	Specificity	
MLP	XGBoost	2	0.91 ± 01.19	0.91 ± 01.19	0.91 ± 01.19	0.91 ± 01.19	0.91 ± 01.19	0.592
Extra Tree	Random Forest	5	0.88 ± 01.17	0.88 ± 01.17	0.88 ± 01.17	0.88 ± 01.17	0.88 ± 01.17	0.406
Random Forest	XGBoost	2	0.87 ± 01.17	0.87 ± 01.17	0.87 ± 01.17	0.87 ± 01.17	0.87 ± 01.17	0.412
KNN	XGBoost	2	0.87 ± 01.17	0.87 ± 01.17	0.87 ± 01.17	0.87 ± 01.17	0.87 ± 01.17	0.464
SVM	XGBoost	2	0.86 ± 01.16	0.86 ± 01.16	0.86 ± 01.16	0.86 ± 01.16	0.86 ± 01.16	0.456
Gradient Boost	XGBoost	2	0.84 ± 01.15	0.84 ± 01.15	0.84 ± 01.15	0.85 ± 01.15	0.84 ± 01.15	0.492
XGBoost	Random Forest	5	0.84 ± 01.15	0.84 ± 01.15	0.84 ± 01.15	0.84 ± 01.15	0.84 ± 01.15	0.426
Logistic Regression	Random Forest	2	0.81 ± 01.13	0.81 ± 01.13	0.81 ± 01.13	0.81 ± 01.13	0.81 ± 01.13	0.532
LDA	Random Forest	9	0.78 ± 01.11	0.78 ± 01.11	0.78 ± 01.11	0.78 ± 01.11	0.78 ± 01.11	0.406
AdaBoost	Random Forest	3	0.68 ± 01.03	0.68 ± 01.03	0.68 ± 01.03	0.70 ± 01.05	0.68 ± 01.03	0.492

**Table 4:** A detailed summary of the performance metric for the best performing combination.

Top Combination of Classifier, Feature Selection, # of Feature	Class	Accuracy	Precision	Sensitivity	F1-Score	Specificity	Inference time (ms)
MLP Classifier XGBoost Feature Selection Technique Top 2 Features	Class 1	0.91 ± 02.49	0.96 ± 02.56	0.95 ± 02.54	0.95 ± 02.55	0.90 ± 02.47	0.592
	Class 2	0.91 ± 03.10	0.86 ± 03.02	0.89 ± 03.07	0.88 ± 03.05	0.91 ± 03.11	
	Class 3	0.91 ± 03.52	0.83 ± 03.36	0.86 ± 03.41	0.84 ± 03.38	0.92 ± 03.53	
	Class 4	0.91 ± 03.06	0.80 ± 02.87	0.86 ± 02.98	0.83 ± 02.93	0.92 ± 03.07	
	Class 5	0.91 ± 01.99	0.98 ± 02.07	0.93 ± 02.02	0.95 ± 02.04	0.90 ± 01.98	
	Overall	0.91 ± 01.19	0.91 ± 01.19	0.91 ± 01.19	0.91 ± 1.19	0.91 ± 01.19	

**Table 5:** Comparison with similar studies.

Studies	Reported Approach	Approach Results
Cruz et al. in [24]	A shallow CNN model named DFTNet was developed to classify using thermogram images	94.57% F1-score for 10 folds with an unconventional approach of taking 2 different classes in each fold and reporting the average of the 10 folds The authors have computed the 5-fold cross-validation results using DFTNet for the original thermogram (68.96% F1-score), Gamma-enhanced thermogram (68.57% F1-score), AHE-enhanced thermogram (67.69% F1-score)
Khandakar et al. [23]	Transfer learning using MobileNetV2 and image enhancement to classify thermograms into control and diabetic	A comparatively shallow CNN model, MobilenetV2 achieved an F1 score of ∼95% for a two-feet thermogram image-based classification, and the AdaBoost Classifier used 10 features and achieved an F1 score of 97%
This study	MLP classifier using 2 features extracted from the thermogram	91.18% F1-score for 5-fold cross-validation for 5 class-classification

## Data Availability

Not applicable.

## Supplementary Materials

The following supporting information can be downloaded at: https://www.mdpi.com/article/10.3390/s22051793/s1, Figure S1: Heat map of the correlation using 38 features (A), and 28 features after removing features with more than 95% correlation (B).
