# Supplementary material for: Thermal Change Index-Based Diabetic Foot Thermogram Image Classification Using Machine Learning Techniques
Source: Sensors (Basel). 2022 Feb 24;22(5):1793. doi: 10.3390/s22051793 (PMC8915003; doi:10.3390/s22051793)
Supplement: Supplementary file 1 [file sensors-22-01793-s001.zip › sensors-1570395-supplementary.pdf]

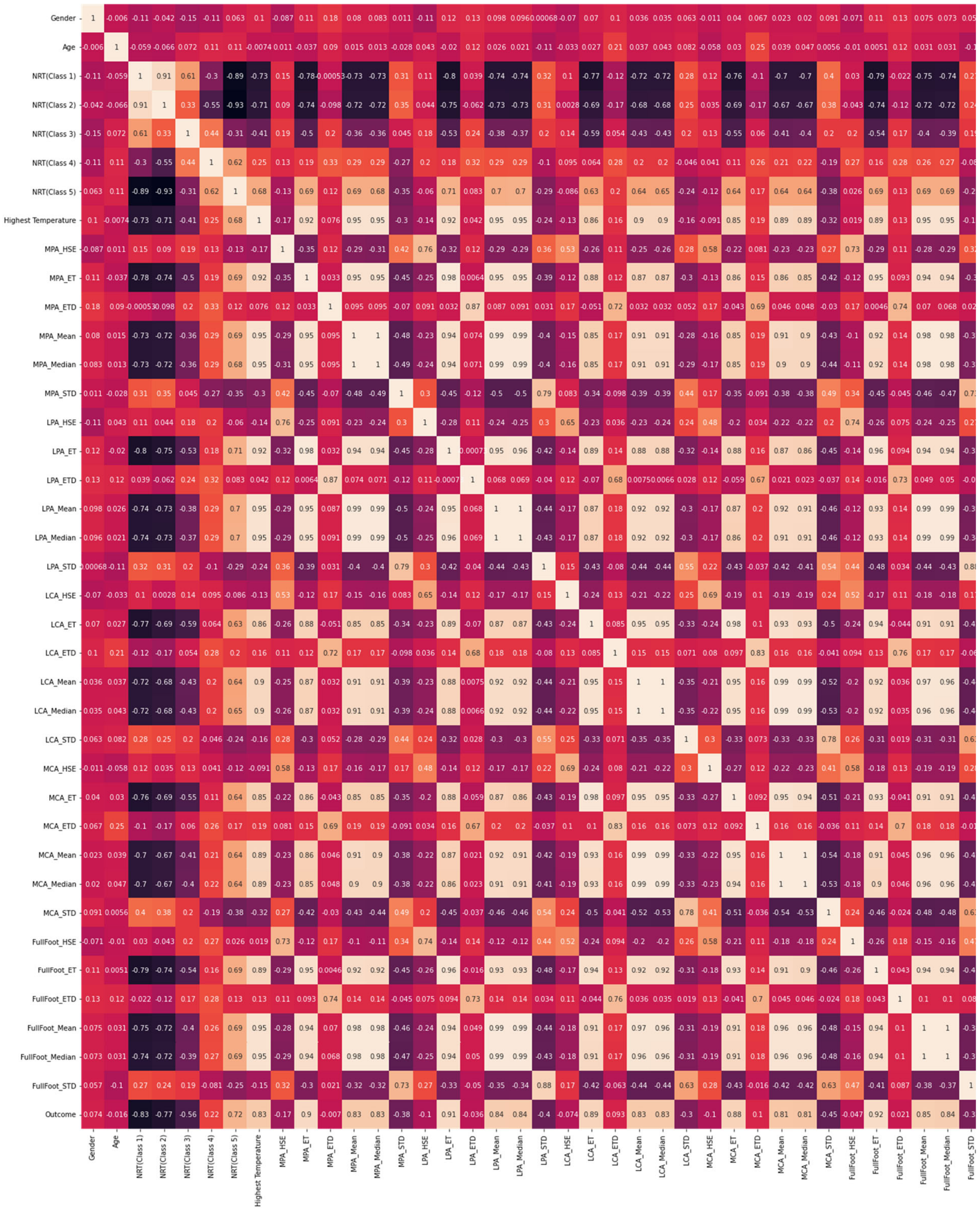
Gender
Age
NRT(Class 1)
NRT(Class 2)
NRT(Class 3)
NRT(Class 4)
NRT(Class 5)
Highest Temperature
MPA_HSE
MPA_ET
MPA_ETD
MPA_Mean
MPA_Median
MPA_STD
LPA_HSE
LPA_ET
LPA_ETD
LPA_Mean
LPA_Median
LPA_STD
LCA_HSE
LCA_ET
LCA_ETD
LCA_Mean
LCA_Median
LCA_STD
MCA_HSE
MCA_ET
MCA_ETD
MCA_Mean
MCA_Median
MCA_STD
FullFoot_HSE
FullFoot_ET
FullFoot_ETD
FullFoot_Mean
FullFoot_Median
FullFoot_STD
Outcome

(A)

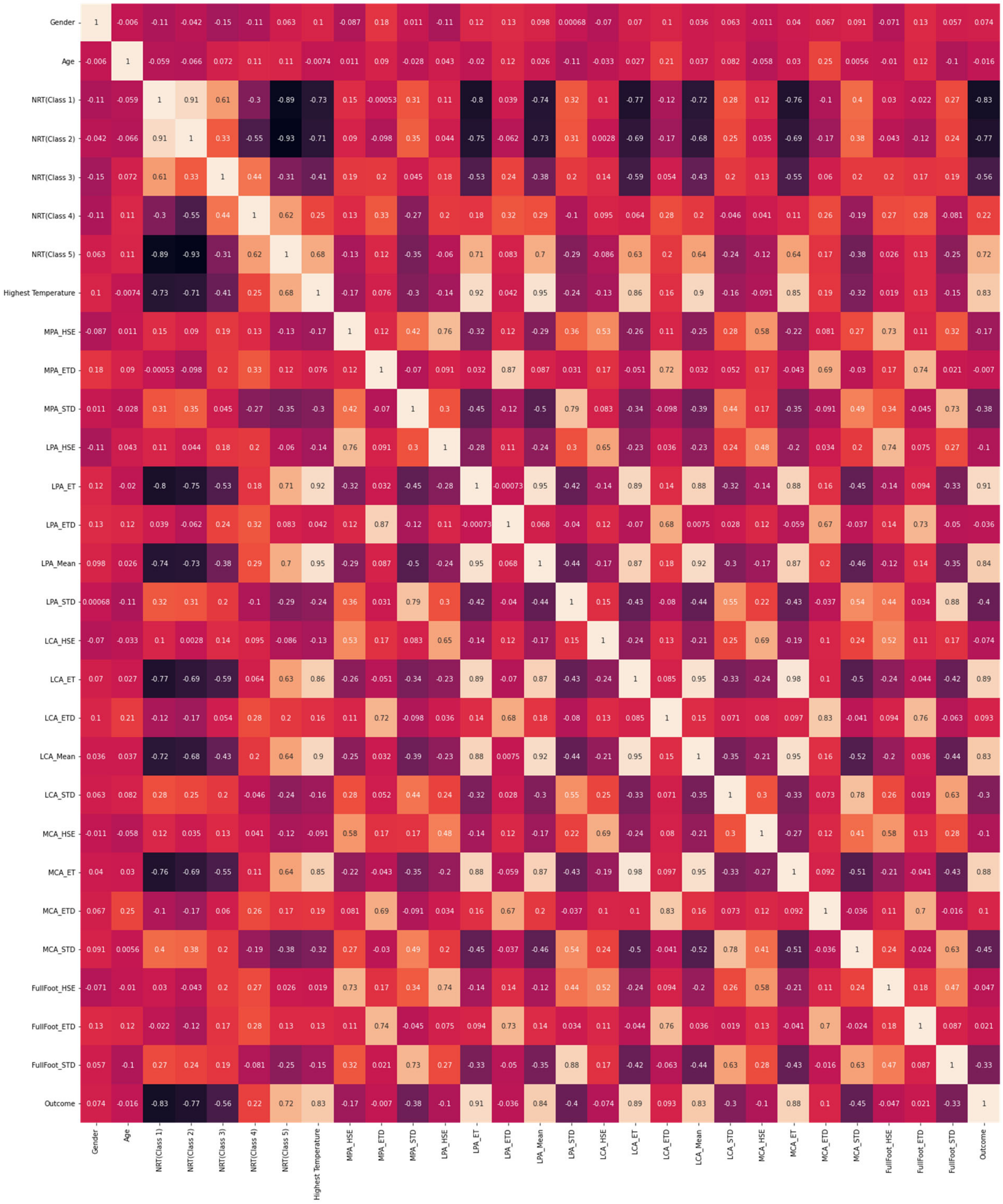

(**B**)

Figure S1. Heat map of the correlation using 38 features (**A**), and 28 features after removing features with more than 95% correlation (**B**).
